# A Rare Isolated Hyoid Bone Fracture: A Case Report and Review of the Literature

**DOI:** 10.7759/cureus.37165

**Published:** 2023-04-05

**Authors:** Bashair M Alwasiyah, Zuhair H Aljehani, Nouran H Farag

**Affiliations:** 1 Department of Otolaryngology - Head and Neck Surgery, King Fahad General Hospital, Jeddah, SAU; 2 Faculty of Medicine, King Abdulaziz University, Jeddah, SAU

**Keywords:** airway compromise, head and neck trauma, maxillofacial, blunt trauma, hyoid bone fracture

## Abstract

Isolated hyoid bone fractures are rare and account for a small percentage of all head and neck fractures. The anatomic location of the hyoid bone, which is between the jaw and the cervical spine, is its most essential protective mechanism. In addition to the anatomic protection provided by the mandible, the fusion of the hyoid's bone pieces and the bone’s mobile capacity in all directions are other protective factors contributing to the rarity of these fractures. However, this defense mechanism can get compromised upon exposure to blunt traumas and hyperextension injuries.

Injury to the neck by blunt trauma can induce fast deterioration, and a missed or delayed diagnosis can result in morbidity and fatality. The importance of early diagnosis and suggested management options are further discussed. We herein report an unusual case of an isolated hyoid bone fracture in a 26-year-old man who was hit by a car while crossing the street. The patient was otherwise asymptomatic and vitally stable so he was managed successfully by conservative management only.

## Introduction

Hyoid bone fractures are uncommon and rarely reported as an isolated entity [1,2]. They account for 0.002% of all head and neck fractures, with 1.15% being the highest incidence. What mainly contributes to the infrequency of hyoid bone fractures is its protected anatomic location under the mandible, as well as the fusion of the hyoid bone’s pieces and the bone’s capacity to move in all directions [3-5]. However, it is worth mentioning that this protection can be distorted by hyperextension injuries and blunt traumas.

Injury to the hyoid bone can be overlooked if there are other life-threatening injuries present or if the patient is asymptomatic. This poses a challenge in picking up these subtle fractures when patients first present to the emergency department (ED).

In this case report, we present a single hyoid bone fracture induced by blunt trauma in a pedestrian versus motor vehicle accident (PVMVA) along with a full description of the injury and treatment options.

## Case presentation

We hereby report a case of a 26-year-old male who presented to the ED as a case of a pedestrian car accident while he was crossing the street. The patient reported that he was hit by a car from his back and fell on the left side of his body. No loss of consciousness occurred. On presentation, the patient's vital signs were within the normal range; blood pressure was 120/67 millimeters of mercury, temperature was 37.1 degree Celsius, heart rate was 88 beats per minute, respiratory rate was 18 breaths per minute, and oxygen saturation was 100% on room air. The patient only complained of mild anterior neck pain.

On examination, Glasgow Coma Scale (GCS) was 15/15. There were multiple abrasions and lacerations noticed on his face, in addition to a small cut wound on his left eyebrow. On inspection of the neck, there was no swelling, wounds, lacerations, abrasions, or any redness noted. There was only mild neck tenderness on palpation. Bedside flexible nasolaryngoscopy was done and showed a clear patent airway with no signs of obstruction, no masses or lacerations in the larynx, and mobile vocal folds bilaterally. The remaining physical examination was normal.

**Figure 1:**
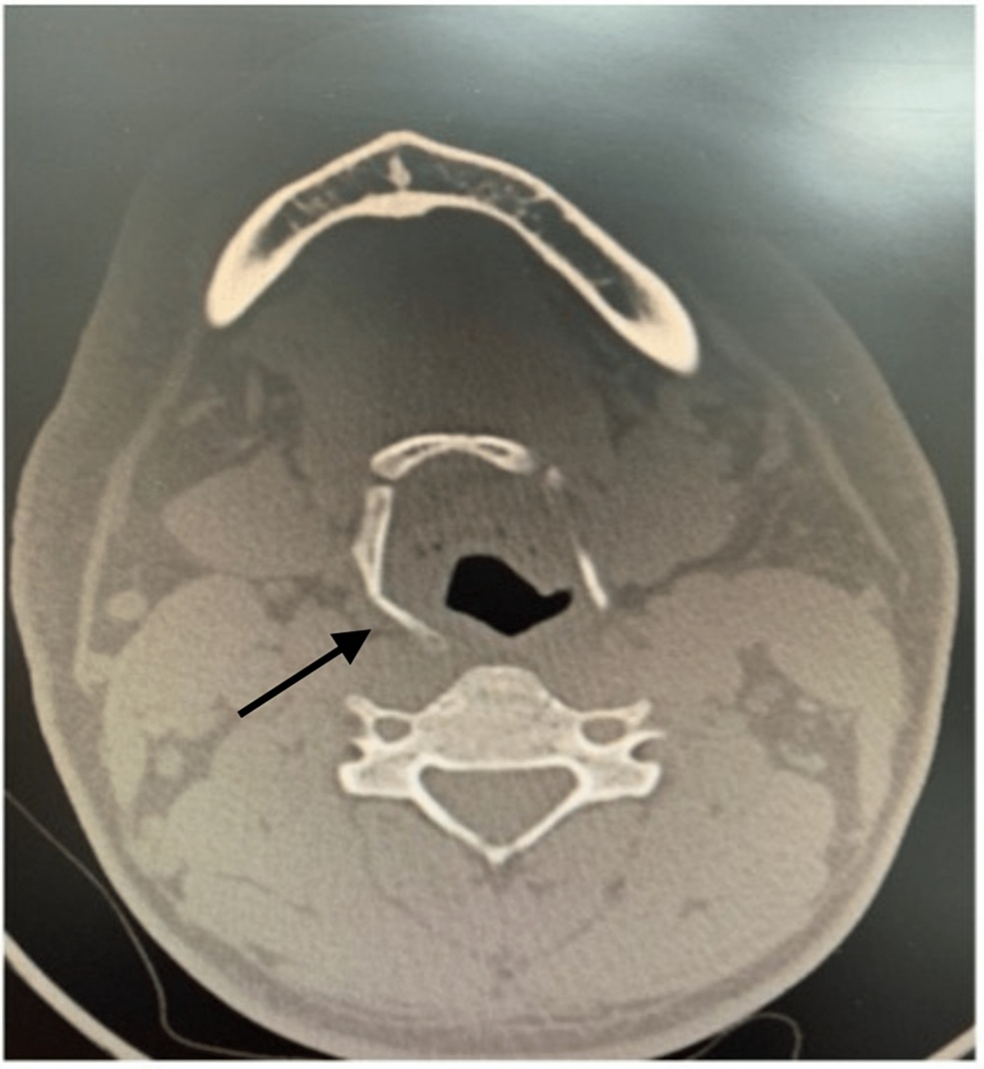
Fracture of the right distal aspect of the greater horn of the hyoid bone (arrow)

After that, a neck computed tomography (CT) scan was done and a hyoid bone fracture was established (Figure 1). The patient was monitored for 24 hours in the ED for any airway obstruction. During observation, analgesics and steroids were given. He was tolerating orally, hence was on a soft diet. There was no neck edema, subcutaneous emphysema, or compromised airways during the physical examination. After 24 hours of observation in the ED, his vital signs remained stable, with no active complaints. He was discharged with all red flags explained in addition to when he needs to come back to ED. A follow-up appointment at the Otolaryngology-Head and Neck clinic within one week was given.

## Discussion

The hyoid bone is a solitary horseshoe-shaped bone in the front of the neck, made up of a body and two bigger and two lesser horns [3]. It is located between the third and fourth cervical vertebrae. It is a dangling styloid protrusion that supports tongue movement in front of the cervical spine, right below the protruding section of the mandible and just above the thyroid cartilage. The hyoid bone is protected by these structures from direct impact [6,7]. Although it does not directly make a joint, the hyoid is a sensitive bone to which the cervical and tongue muscles are attached [2,8]. The hyoid’s omnidirectional mobile capacity, between the jaw and cervical spine, contributes to the less likelihood of its fracture [9]. However, during cervical hyperextension, protection of the hyoid from surrounding bones reduces, and thereby, fractures can happen even in the absence of direct trauma [5,10]. In this position, many of the muscles attached to the hyoid bone are tense, restricting the hyoid's mobility and consequently its ability to absorb stresses. On the contrary, when patients are in a relaxed position, the anatomic linkages of the hyoid bone make it extremely difficult for an isolated fracture to occur.

Different causes for hyoid bone fractures have been reported in the literature within different settings. According to Erdogan B et al. and Chowdhury R et al., the most common causes of hyoid injuries are strangulation and hanging [8,11]. Similarly, Olmstead et al. reported in their case series a case of hyoid fracture in a 34-year-old male who attempted suicide by hanging [12]. Even though killing tactics that impact and imprison the neck have been used since the dawn of civilization, high-energy blunt neck traumas, like that caused by explosions, car accidents, or martial arts contests, have grown more common in recent years and can induce fast deterioration [13]. A case of hyoid bone fracture was reported by Porr J et al. in Canada for a 13-year-old male taekwondo athlete after experiencing a kick in the anterior neck during a match [3]. Boussaid et al. reported a case of hyoid bone fracture found incidentally during an autopsy of a woman who was found dead face down in a six-meter-deep well [2].

Fractures of the hyoid bone are frequently combined with injuries to the mandible, cervical spine, larynx, and throat because of its proximity to the surrounding structures. Because these injuries are more urgent, hyoid fractures may not be recognized right away [12]. Missed or delayed diagnosis in such cases may lead to increased morbidity and fatality.

Patients with hyoid bone fractures may have a wide range of symptoms [11,12]. The most prevalent of these are pain in the anterior neck, pain with head rotation, and symptoms associated with swallowing such as dysphagia or odynophagia. However, the most dangerous symptoms are those associated with airway compromise. Typical clinical findings include tenderness in the anterior neck when palpated, apparent swelling in the neck, and inability to fully rotate the head [3]. In our reported case, the patient was asymptomatic, unlike other reported cases in the literature. According to Olmstead et al., the patient reported having a hyoid fracture secondary to a failed suicidal hanging attempt and experienced neck pain, hoarseness, and dysphagia [12]. Another case reported by Porr et al. mentioned that the patient, a taekwondo athlete who got kicked in the neck during a match, experienced symptoms related to the airway, mainly in the form of dyspnea, wheezing, and coughing [3]. Rifai et al. reported two cases of hyoid bone fracture whose main presentation was severe throat pain and eventually deteriorated and developed respiratory distress [14]. On standard fiber-optic endoscopy, common findings include pharyngeal lacerations, hematomas, edema, and portions of the hyoid bone projecting through the pharyngeal mucosa. Several strategies for better viewing the pyriform sinuses and post-cricoid zones have been documented. Modified Valsalva maneuver, trumpet maneuver, and skin traction are some of the methods used [5].

A hyoid bone fracture can be diagnosed by physical examination, cervical radiography, a CT scan, or direct laryngoscopy [3]. However, these fractures may be difficult to detect on a frontal cervical radiograph and are subtle on lateral cervical radiographs [7]. So, a cervical CT scan should be performed to rule them out [7,9]. During a radiological assessment, other head and neck injuries that should be ruled out are mandibular fractures, facial fractures, thyroid cartilage fractures, pharyngeal trauma, laryngeal lacerations, cervical spine injuries, vascular trauma, and external carotid artery pseudo-aneurysms [6-8]. The most significant diagnostic tool for evaluating the bone components of the neck is a CT scan, which can also detect the presence of laryngeal trauma [13]. In this case, a neck CT scan was done, and it demonstrated a displaced hyoid bone fracture with edema of the underlying soft tissues with no evidence of an airway compromise.

The focus of this case is the management of a patient with an uncommon, isolated fracture of the right larger horn of the hyoid bone in a pedestrian versus motor vehicle accident (PVMVA).

The treatment of a hyoid bone fracture is mostly determined by the severity of the symptoms. For closed or asymptomatic fractures, conservative therapy is usually sufficient, and surgical intervention is rarely required [6-8]. Although most of these fractures are managed conservatively and without complications, they are potentially life-threatening and can lead to serious respiratory impairment [15]. In cases of severe airway compromise, tracheal intubation or surgical tracheostomy is required to secure the airway [7]. According to Erdogan B et al., close observation for at least 24 hours is essential in asymptomatic individuals to assess airway security [8]. Cutuk A et al. reported two cases of football athletes who sustained direct traumas during the games, which lead to hyoid fractures. Both patients experienced symptoms involving voice quality and swallowing but were otherwise hemodynamically stable. They were successfully managed conservatively and observed for 24-48 hours [7]. Analgesics, fixation with a soft neck rest, and a soft or liquid diet may be used in conjunction with conservative therapy [3]. On the contrary, open or symptomatic hyoid bone fractures associated with a pharyngeal laceration are an indication for surgical exploration [8,14]. In our reported case, during the patient's observation in the ED, his presenting symptoms did not increase or produce acute airway compromise. The patient was continuously monitored with his head elevated, analgesics and steroids administered, and a soft diet initiated.

Other cases of hyoid bone fractures reported in the literature by Dalati T et al. and Keerthi R et al. recommended that all patients be monitored for 48-72 hours because even asymptomatic individuals are at risk of developing hemoptysis, edema, ecchymosis, and spasm, which may require tracheostomy and retro-pharyngeal drainage [5,6]. However, due to the rarity of isolated hyoid bone fractures, there are no established guidelines for intervention or standard timing for observation. Treatment is usually determined by whether there is laryngeal or pharyngeal perforation. In cases where there is no perforation, conservative treatment is recommended [5,7]. To control ecchymosis and pain, ice and analgesics may be used [3]. When there is laryngeal or pharyngeal perforation, removal of the fractured hyoid and any fragments, as well as suturing and fixation, may be warranted. In cases of soft tissue compromise, a risk of infection exists so body temperature should be monitored [5,6,14]. According to Cutuk et al., a liquid diet, voice rest, and possibly a nasogastric feeding tube may be used as part of conservative therapy. In our reported case, the management was similar to that previously described by Cutuk et al. for hyoid bone fractures without laryngeal laceration [7]. Because of the patient's presenting symptoms, an ambulance was dispatched for concern of worsening dyspnea and probable suffocation.

## Conclusions

Although rare, hyoid bone fractures could be life-threatening, they do not require surgical intervention unless the patient has a compromised airway, is symptomatic, or becomes symptomatic during the hospital stay. Observation has traditionally been the primary management modality, with a 24-72-hour hospital stay followed by a two-week post-injury evaluation after discharge.
